# Induction of Cell Cycle Arrest and Apoptotic Response of Head and Neck Squamous Carcinoma Cells (Detroit 562) by Caffeic Acid and Caffeic Acid Phenethyl Ester Derivative

**DOI:** 10.1155/2017/6793456

**Published:** 2017-01-12

**Authors:** Arkadiusz Dziedzic, Robert Kubina, Agata Kabała-Dzik, Marta Tanasiewicz

**Affiliations:** ^1^Department of Conservative Dentistry with Endodontics, School of Medicine with the Division of Dentistry, Medical University of Silesia in Katowice, Pl. Akademicki 17, 41-902 Bytom, Poland; ^2^Department of Pathology, School of Pharmacy and Division of Laboratory Medicine in Sosnowiec, Medical University of Silesia in Katowice, ul. Ostrogórska 30, 41-200 Sosnowiec, Poland

## Abstract

Natural polyphenols have been observed to possess antiproliferative properties. The effects, including apoptotic potential of bioactive phenolic compounds, caffeic acid (CA) and its derivative caffeic acid phenethyl ester (CAPE), on cell proliferation and apoptosis in human head and neck squamous carcinoma cells (HNSCC) line (Detroit 562) were investigated and compared. Cancer cells apoptosis rates and cell cycle arrests were analysed by flow cytometry. Exposure to CA and CAPE was found to result in a dose-dependent decrease in the viability of Detroit 562 cells at different levels. CA/CAPE treatment did significantly affect the viability of Detroit 562 cells (MTT results). CAPE-mediated loss of viability occurred at lower doses and was more pronounced, with the concentrations which inhibit the growth of cells by 50% estimated at 201.43 *μ*M (CA) and 83.25 *μ*M (CAPE). Dead Cell Assay with Annexin V labelling demonstrated that CA and CAPE treatment of Detroit 562 cells resulted in an induction of apoptosis at 50 *μ*M and 100 *μ*M doses. The rise of mainly late apoptosis was observed for 100 *μ*M dose and CA/CAPE treatment did affect the distribution of cells in G0/G1 phase. A combination of different phenolic compounds, potentially with chemotherapeutics, could be considered as an anticancer drug.

## 1. Introduction

Polyphenols, the main constituents of honey bee hive product propolis, are well known to inhibit cell proliferation and induce cell death in human cancer cells [1–3]. The biological activities of propolis are mainly attributed to caffeic acid, cinnamic acid, phenethyl esters, p-coumaric acid, artepillin C, galangin, cardanol, baccarin, chrysin, and other ingredients which possess oxyradical scavenging properties [2–4]. Recent evidence indicates that polyphenols and flavonoids are responsible for an induction of apoptosis and cell cycle inhibition, antiangiogenesis, suppression of matrix metalloproteinases, prevention of metastasis, and augmentation of the effects caused by chemotherapy [3–7]. These compounds are intracellularly metabolized via multiple pathways targeting distinct molecules and exhibiting wide spectrum of cellular cytotoxicity in different cancer types. More specifically, propolis constituents, including phenolic acids effect tumor cells through apoptosis, cell cycle arrest, and cytostatic activity, induced endoplasmic reticulum stress, and caspase activity also reduced mitochondrial membrane potential [8–11]. However, the precise mechanisms by which propolis constituents, caffeic acid and its caffeic acid phenethyl ester, activate apoptosis in human cancer cells still remain uncertain and inconsistent.

Organic phenolic compounds including caffeic acid (CA) and its derivative caffeic acid phenethyl ester (CAPE) are known to be highly bioactive components extracted from honeybee hive propolis [12, 13]. Recent studies indicate that they exhibit cytotoxic, antiproliferative [14, 15], anti-inflammatory, immunomodulatory [16, 17], antioxidative [17–20], and antibacterial properties [17, 21]. CA and particularly CAPE treatment suppresses proliferation, survival, and invasion of human malignant metaplastic cells, including oral cancer cells [14, 15, 22–25]. Our recent study demonstrated that caffeic acid is able to attenuate the viability and migration rate of oral cancer SCC-25 cells [26]. To the best of our knowledge, there are limited previous studies comparing the growth inhibition of human head and neck squamous carcinoma cells by different polyphenols and/or flavonoids. According to available data, tea catechins are the only flavonoids used in clinical studies on oral cancer [27].

Above 90% of oral and head and neck malignancies are classified histologically as squamous cell carcinoma (SCC) [28, 29]. Squamous cell carcinoma is the most lethal head and neck cancer and, according to epidemiological data, it belongs to the sixth most common epithelial malignancies worldwide. Low survival rate of patients is linked to regional lymph node metastases, poor response to current therapeutic drugs, and local relapse [30]. Although research development in oral and cancer therapy over the recent decades is undoubtedly significant, treatment outcome of HNSCC may not be successful for a significant group of patients, resulting in cancer recurrence and progression, with a decreased overall survival rate. Bioactivity of propolis and plants phytochemicals constituents, including CA and CAPE compounds, is directly attributed to their chemopreventive potential in oral squamous cell carcinoma and generally in human oral carcinogenesis [31–33]. The synergistic and/or additive effects of common components, identifiable in propolis, plants, and vegetable/fruits, are responsible for the chemoprotective action of “healthy organic food” and may play important role in oral and pharyngeal cancer prevention [34, 35].

The current in vitro study has been arranged to investigate the cytotoxic effects of two bioactive phenolic constituents of propolis: caffeic acid and caffeic acid phenethyl ester on the viability, apoptosis, and cell cycle arrest of head and neck (HNSCC) squamous carcinoma cells Detroit 562 line.

## 2. Material and Methods

### 2.1. Cell Line Culture Conditions and Reagents

Detroit 562 human squamous carcinoma cell line originating from pharynx primary location was used in the present study and purchased from the European Collection of Authenticated Cell Cultures (ECACC, Salisbury, UK). Detroit 562 HNSCC cells were seeded on 6-well microplates and were cultured in standard culture medium (EMEM; Eagle's Minimum Essential Medium) containing 10% fetal bovine serum (FBS; Pasching, Austria) and 1% penicillin-streptomycin (PAA Laboratories GmbH, Pasching, Austria) at 37°C in 5% CO_2_ in air (CO_2_ incubator, Heraeus Instruments, Hanau, Germany). Additionally, cells were cultured with 100 *μ*g/mL streptomycin, 100 IU/mL penicillin, and 0.25 *μ*L/mL amphotericin B at 37°C in a 5% CO_2_ atmosphere. Reagents were purchased from PAA Laboratories GmbH (Pasching, Austria); caffeic acid and caffeic acid phenethyl ester were purchased from Sigma (St. Louis, MO, USA). Muse™ Annexin V and Dead Cell kit were purchased from Millipore (Billerica, MA, USA).

### 2.2. Cell Viability/Proliferation Assay

Detroit 562 HNSCC cells proliferation was measured by the (4,5-dimethylthiazol-2-yl)-2,5 diphenyltetrazolium bromide (MTT) assay. Cells were seeded on 96-well microplates at 5 × 10^3^ cells/well and left for 48 h in to enable them to attach to the culture medium. Culture medium was decanted and to each well a culture medium-containing CA or CAPE with concentration from 100 to 5 *μ*M was added and left for 24 or 48 h. Next, cell medium was decanted and 10 *μ*L of MTT solution (5 mg/mL MTT in phosphate-buffered saline (PBS)) was added and left for 3 h. Formed formazan crystals were dissolved in DMSO.

**Figure 1:**
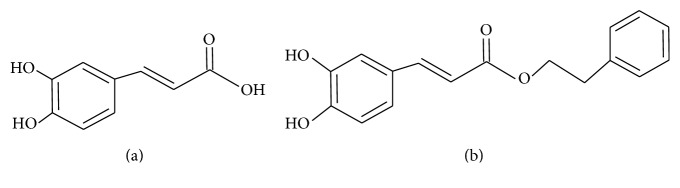
Chemical formulas of bioactive phenolic compounds: caffeic acid (CA) classified as hydroxycinnamic acid (a) and CAPE (b). Caffeic acid comprises both phenolic and acrylic functional groups.

Live cells appeared purple in colour in response to MTT. The investigated substances CA and CAPE were applied to monolayer cultures of Detroit 562 human head and neck cancer cells at the final concentrations from 5 to 100 *μ*M, except for the control cells, to which nutrient medium was applied. One hundred microliters of supernatant was transferred to a 96-well plate and cell viability was determined using Elx800 microplate reader (Bio-Tek Instruments Inc., Winooski, VT, USA), by measuring a spectrometric absorbance at 570 nm. The half and quarter maximal Inhibitory Concentration (IC_50_, IC_25_) value of the CA and CAPE was determined for monolayer cells. The chemical structure of CA and CAPE is presented in Figures 1(a) and 1(b), respectively.

### 2.3. Cell Apoptosis Assay: Analysis of Viability and Cell Death Using Flow Cytometry

Detroit 562 cell apoptosis and dead cells, including the percentage of apoptotic cells, were assayed using the multifunctional Muse Annexin V and Dead Cell kit (Millipore, Billerica, MA, USA) according to the user's guide and the manufacturer's instructions. Briefly, after treatment with CA and CAPE, Detroit 562 cancer cells were harvested with trypsin-EDTA and washed twice in PBS. Fresh medium-containing serum was added to each well so final concentration was 1 × 10^5^ cells/mL. Staining protocol included warming the Muse Annexin V and Dead Cell Reagent to room temperature, addition of 100 *μ*L of cells in suspension to each tube, addition of 100 *μ*L of the Muse Annexin V and Dead Cell Reagent to each tube, and mixing thoroughly by vortexing at a medium speed for 5 seconds. Cells were resuspended in PBS with 1% FBS, mixed with the Muse Annexin V and Dead Cell reagent. Samples were incubated for 20 minutes at room temperature in the dark. The percentage of apoptotic cells was analyzed by flow cytometry using Muse Cell Analyzer (Millipore, Billerica, MA, USA) system and were expressed as percentage of apoptotic cells and standard deviation bars represent SD. As a negative control we used pure medium with FBS serum and as a positive control the medium with paclitaxel addition at concentration 100 nM.

### 2.4. Flow Cytometry Analysis of Cell Cycle Detroit 562 Arrest

Detroit 562 cells were seeded in 4-well plates and incubated with medium containing 10% FBS at 37°C. After treatment with CA and CAPE cell samples were transferred to 15 mL conical tube and the minimum number of cells for fixation in a tube was amounted at 1 × 10^6^ cells. Samples collected after 24 h and 48 h were gently centrifuged for 5 min at 1500 rpm and washed in PBS. Obtained pellets were fixed in chilled 70% ethanol. Detroit 562 cells were kept in −20°C for 7 days until cell cycle was assayed. After ethanol removal cells were suspended in 0.25 mL PBS per 5 × 10^5^ cells and warmed up to 37°C. Cell pellet was resuspended in 200 *μ*L of Muse Cell Cycle Reagent, incubated for 30 minutes at room temperature, protected from light, and cell suspension was transferred to a 1.5 mL microcentrifuge tube prior to analysis on Muse Cell Analyzer. Cell cycle was assayed by fluorescence-activated cell sorting analysis using a Muse Cell Analyzer (Merck, Millipore, Billerica, MA, USA) with the configuration of 532 nm green laser line, three detection channels, and microcapillary 100 *μ*L round bore.

### 2.5. Statistical Analysis

Data are presented as means ± standard deviation (SD) and were analyzed by nonparametric methods using the Statistica 9.0v (StatSoft, Tulsa, OK, USA) computer-based statistics programs. Statistical differences between means were evaluated by Friedman ANOVA variance analysis followed by post hoc Dunn's test and Wilcoxon test. The value of *p* < 0.05 was considered to be significant (*∗*), *p* < 0.01 and *p* < 0.001 as highly significant (*∗∗* and *∗∗∗*, resp.). The results were obtained from three separate experiments performed in quadruplicates (*n* = 12) for cytotoxicity. The experimental means were compared to the means of untreated cells harvested in a parallel manner. IC_25_ and IC_50_ values were calculated from the corresponding concentration inhibition curves according to plotted data presentation based on representative graphs.

## 3. Results

The study was aimed at comparison of the influence of two common phenolic compounds, constituents of propolis: caffeic acid and caffeic acid phenethyl ester on inhibition of the proliferation, viability and growth of squamous carcinoma cells, as recent reports have confirmed the beneficial effect of propolis-induced cellular stress on selected tumor cells [23–26]. The cellular effect on the HNSCC cell line Detroit 562 was investigated in vitro with the use of MTT assay in a microculture system using various incubation concentrations. Cytotoxic efficacy of CA and CAPE was expressed as the percentage of viable HNSCC Detroit 562 carcinoma cells at different concentrations of CA/CAPE with regard to the unexposed cells. The half maximal Inhibitory Concentration (IC_50_) was defined as the CA/CAPE concentration value which inhibits the viability of Detroit 562 HNSCC cells in culture by 50% compared to the untreated cells (control). The quarter maximal Inhibitory Concentration (IC_25_) was defined as the CA/CAPE concentration value which inhibits the viability of Detroit 562 HNSCC cells in culture by 25% compared to the untreated cells (control). IC values were extrapolated from cell viability-CA/CAPE concentration curves. To establish the concentration required to cause effects of 50% growth inhibition in Detroit 562 cells after 24 h and 48 h, a log viability-log dose curve was plotted.

### 3.1. High Concentrations of CA and CAPE Decrease of Head and Neck Detroit 562 Cell Line Viability and Mitochondrial Function

**Figure 2:**
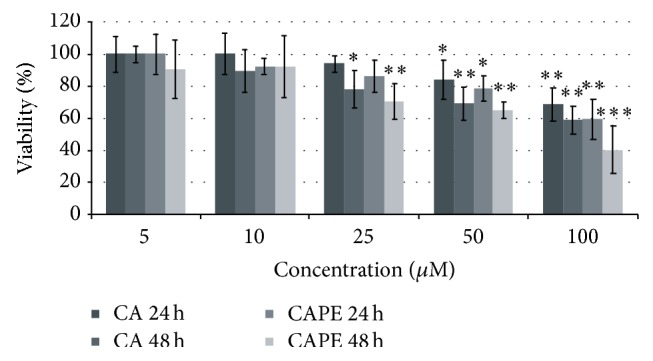
Cytotoxic effects of CA and CAPE at concentrations 5–100 *μ*M on Detroit 562 cancer cells. These effects are highly concentration-dependent. The percentage of cell death measured by MTT cytotoxicity assay. MTT values represent mean ± SD of three independent cytotoxicity experiments performed in quadruplicate (*n* = 12). The lower concentration of CAPE (25 *μ*M) produced similar killing effect on Detroit 562 cells as 50 *μ*M concentration of CA. Mean cytotoxicity between different concentrations alone was statistically significant above the concentration of 25 *μ*M (^*∗*^*p* < 0.05 and ^*∗∗*^*p* < 0.01, ANOVA Friedman ANOVA test, Wilcoxon test). CA and CAPE at concentrations range of 25–100 *μ*M induce cytotoxic effects on HNSCC carcinoma cells in a dose-dependent manner and displayed a time-dependent influence during 24 and 48 h of experiment. On the contrary, CA/CAPE concentrations within the range 5–10 *μ*M did not alter markedly the Detroit 562 cells' viability and indirect proliferation during 24 h and 48 hours of exposure, reflected by only a slight increase of absorbance. ^*∗∗∗*^*p* value <0.001.

Results of our experiment revealed that the investigated propolis-derived substances at concentrations up to 25 *μ*M exhibit relatively low cytotoxic activity against Detroit 562 cells. As shown in Figure 2, after 24 h/48 h exposure of Detroit 562 cells to 10 *μ*M of CA/CAPE, the cell viability decreased slightly, except for CA/24 h. However, the absorbance value significantly increased and cytotoxicity increased significantly for CA/CAPE concentrations above 25 *μ*M (*p* < 0.05, *p* < 0.01, and *p* < 0.001, depending on time and substance). The overall viability of Detroit 562 cells significantly decreased for CA and CAPE concentrations of 50 *μ*M and 100 *μ*M (*p* < 0.01, *p* < 0.001), with the cell viability reduction between 16% (CA 24 h 50 *μ*M) and 60% (CAPE 48 h 100 *μ*M). For the concentrations of 25 *μ*M and 50 *μ*M of CA and CAPE the cell viability decrease was similar after 48 hours (Figure 2). These findings were enhanced by validating the dose required to inhibit growth of 50% of HNSCC cells (IC_50_) which exhibited a value range 201.43 *μ*M–83.25 *μ*M after 48 h of incubation time. The minimum CA and CAPE concentrations required to cause 25% and 50% cell growth inhibition after 48 h were 31.30 *μ*M (IC_25_, CA), 201.43 *μ*M (IC_50_, CA) and 18.84 *μ*M (IC_25_, CAPE), and 83.25 (IC_50_, CAPE), respectively, while the IC_25_ and IC_50_ values for 24 h of incubation time were much higher: 93.01 *μ*M (IC_25_, CA), 1061.61 *μ*M (IC_50_, CA) and 45.03 *μ*M (IC_25_, CAPE), and 340.95 (IC_50_, CAPE).

### 3.2. Exposure to CA/CAPE Stimulates Cell Apoptosis of Detroit 562 Cells

**Figure 3:**
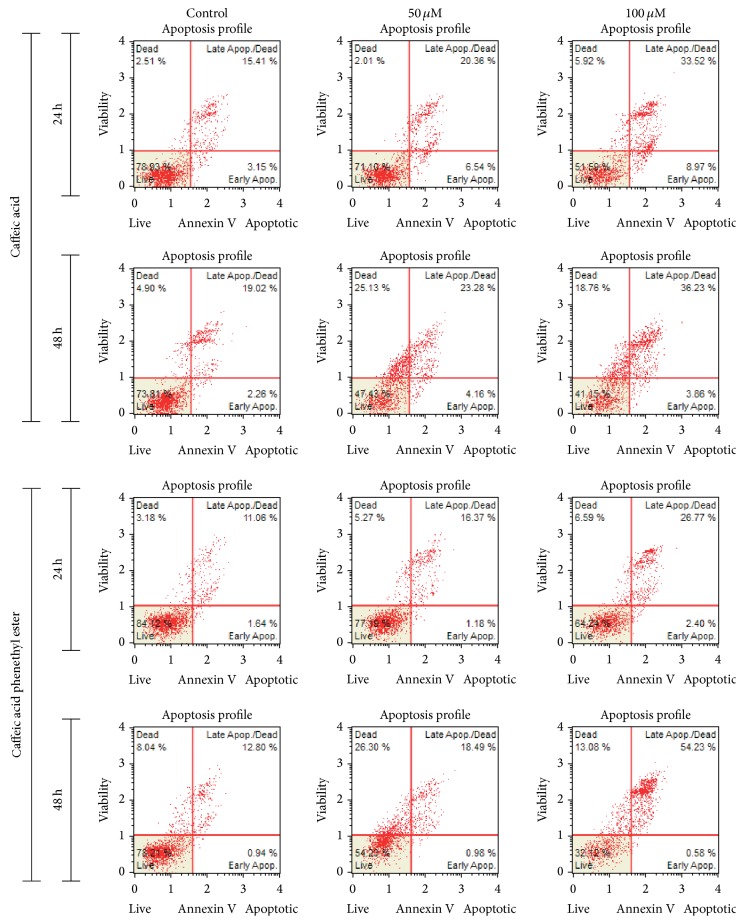
Effect of CA and CAPE substances on Detroit 562 cell apoptosis (representative plots). Early apoptotic cells are shown in the lower-right quadrant of the scatter plot, and live cells are in the lower-left quadrant. Both phenolic compounds CA and CAPE induced apoptosis in a dose-dependent manner as measured by the Muse Annexin V and Dead Cell assay. Flow cytometry was shown to induce apoptotic cell death in the epithelial tumor cells Detroit 562 by mainly early and late apoptosis, which was apparent when the percentage of live cells markedly decreased.

**Figure 4:**
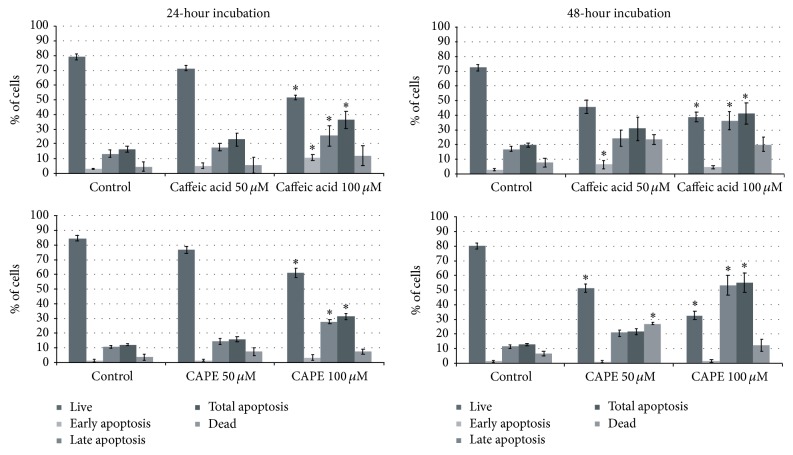
Flow cytometric analysis demonstrated a significant increase in proportion of total apoptotic cells in the NHSCC cells following exposure to mainly CAPE 100 *μ*M and CA 100 *μ*M, also with increased percentage of dead cells. Total apoptotic cells significantly increased following exposure to 100 *μ*M CAPE, compared with 100 *μ*M CA after 48 hours (*p* < 0.05). Specifically, the difference between CA and CAPE for both concentrations 50 *μ*M and 100 *μ*M in the percentage of early apoptotic cells was slight for the time laps 24 h, whereas the difference between them in the percentage of late apoptotic cells was significant for concentration 100 *μ*M (*p* < 0.05). Vertical bars represent the standard deviation of means (SD) (*n* = 3 experiments). ^*∗*^*p* value <0.05.

To investigate the apoptotic effect of CA and CAPE, Detroit 562 cells were treated with both substances for 24 h and 48 h, and apoptotic cells were assessed by staining with Annexin V. To determine whether CA/CAPE treatment results in apoptosis in Detroit 562 HNSCC cells, we used a Muse Annexin V and Dead Cell kit to measure the changes in cell apoptosis after 24 h and 48 h. We observed that both investigated substances induced cell death through apoptosis in Detroit 562 HNSCC cells (Figures 3 and 4). Comparative and similar results were obtained for 24 h and 48 hours. As shown in Figure 4 total apoptotic Detroit 562 cells following exposure to 100 *μ*M CAPE for 24 h and 48 h were significantly increased (31 ± 2.0% and 55 ± 6.71%, resp.) compared with nontreated control (12 ± 0.6%, *p* < 0.05). In particular, the difference between exposure of Detroit 562 cells to 50 and 100 CAPE in the percentage of early apoptotic cells was minimal (1.47% versus 3.49% and 1.12% versus 1.71%, *p* > 0.05), whereas the variation between the cell groups in the percentage of late apoptotic cells was more pronounced for different concentrations and time laps of both CA and CAPE. These data suggest that phenolic compounds such as CA/CAPE suppress cell viability in Detroit 562 cells via apoptotic pathway.

For the highest CA concentration 100 *μ*M, total apoptosis of Detroit 562 cells increased: 36 ± 6.0% (24 h) and 41 ± 7.0% (48 h) compared to 16 ± 2.1% and 19 ± 1.2%, respectively, in controls. For the highest CAPE concentration 100 *μ*M, total apoptosis of Detroit 562 cells increased: 31 ± 2.0% (24 h) and 55 ± 6.7% (48 h) compared, respectively, to 12 ± 0.6% and 13 ± 0.7% in controls (Figure 4). CA-induced and CAPE-induced total apoptosis of Detroit 562 cells for the concentration 50 *μ*M was determined at 22% (24 h) and 30% (48 h) versus 16% (24 h) and 21% (48 h), respectively, for CA and CAPE (Figure 4). The results suggest that the relative apoptosis efficacy (late and total apoptosis) of 100 *μ*M CAPE in Detroit 562 cells after 48 hours is substantially higher compared to 100 *μ*M CA. The apoptotic spectrum of Detroit 562 cells after 24 h of 100 *μ*M CA treatment seems to be roughly an equivalent of 100 *μ*M CAPE exposure. The difference between two time laps, 24 h and 48 h for both concentrations 50 *μ*M and 100 *μ*M, in the percentage of late and total apoptotic cells, was significant for both substances CA and CAPE (*p* < 0.05), whereas the difference between these two time laps in the percentage of early apoptotic cells was slight. Generally, CAPE induced more apoptosis in Detroit 562 cells than did CA after 48 hours and in opposite, CA induced more apoptosis in Detroit 562 cells than did CAPE after 24 hours. The weakest effect was observed in the cells treated with 50 *μ*M of CA for 24 hours.

### 3.3. Effect of Two Concentrations of CA and CAPE on Detroit 562 Cell Cycle Phase Distribution: CA/CAPE Arrests HNSCC Cells at the G0/G1 Phase

**Figure 5:**
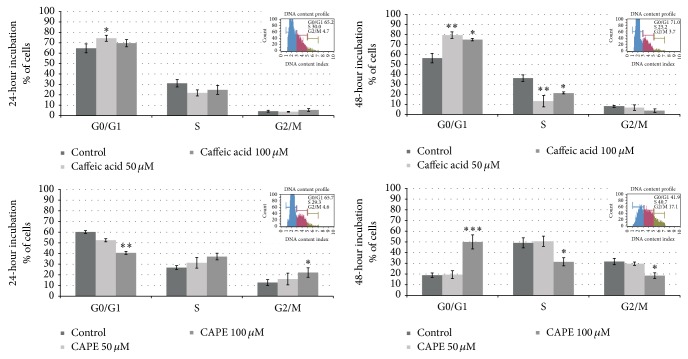
Alterations in the percentage of Detroit 562 cells in G0/G1, S, and G2/M phases of cell cycle are presented as the % of Detroit 562 of three independent experiments (bar graphs). The results show that CAPE at concentration of 100 *μ*M has a mild effect on cell cycle arrest, which is contributing to its anticancer features. Detroit 562 cells exposure to CA and CAPE concentration for 48 h resulted in a cell cycle checkpoint arrest within the G0/G1 phase (^*∗*^*p* < 0.05, ^*∗∗*^*p* < 0.01, and ^*∗∗∗*^*p* < 0.001 independent experiments). Four representative flow cytometric plots (right upper) showing the cell cycle distribution following the Detroit 562 cells treatment with CA and CAPE at 50 and 100 *μ*M for 24 h and 48 h. Cells were stained with Muse Annexin V and Dead Cell kit and were subjected to flow cytometric analysis that collected 10,000 events. The cell cycle distribution within 24 h following exposure of Detroit 562 cells to 50 and 100 *μ*M of CA shows that CA treatment did not markedly affect the distribution of cells among the different phases of the HNSCC cell cycle. However, there was a slight increase in cell numbers in the S phase and G2/M phase when treated with 100 *μ*M CAPE for 24 h (*p* > 0.05 and *p* < 0.01). Treatment with 100 *μ*M CAPE for 48 h resulted in a significant accumulation of cells in the G0/G1 phase for Detroit 562 cell line (*p* < 0.001). Subsequently, cell number in the S phases and G2/M phase was decreased to 31% and 18%, respectively, when exposed to 100 *μ*M CAPE for 48 h (*p* < 0.05 versus control).

Due to the fact that previous studies demonstrated modulation of the HNSCC cell cycle by propolis compounds [14, 22], the CA and CAPE effect on Detroit 562 cell cycle status was examined. The effect of CA and CAPE on the cellular cycle distribution was quantified using flow cytometric analysis and cell cycle progression was examined after treatment with 50 and 100 *μ*M of CA and the same concentrations of CAPE for 24 h and 48 h. As shown in Figure 5, treatment of Detroit 562 cells with CA dose of 50 *μ*M and 100 *μ*M for 48 h h resulted in a significantly higher percentage (80 ± 3.2% and 75 ± 1.4%) of cells in the G0/G1 phase than in the control group (56 ± 4.8%, *p* < 0.05 and *p* < 0.01), with a corresponding reduction in the percentage of cells in the S phase (13 ± 5.6% and 21 ± 1.0%, resp., *p* < 0.05 and *p* < 0.01). More pronounced arrest of G0/G1 phase was observed for 100 *μ*M CAPE (50 ± 6.5%) when cells were treated for 48 h compared to the control (19 ± 2.1%, *p* < 0.001) (Figure 5). These data suggest that inhibition of cell proliferation or induction of cell death in Detroit 562 cancer cells by CA/CAPE is associated mainly with the induction of G0/G1 arrest considering the time laps of 48 hours. The different proliferation rates of Detroit 562 cells exposed to CA/CAPE versus control, untreated cells were partially due to the differences in cell cycle regulation.

As shown in Figure 5, no significant difference between the untreated cells and cells exposed to 50/100 *μ*M CA after 24 hours was observed. However, the percentage of Detroit 562 cells in G0/G1 phase slightly increased up to 74% (CA 50 *μ*M), compared to control (64 ± 4.2%, *p* < 0.05), and the S phase cells decreased (21/24% CA 50/100 *μ*M versus 31 ± 3.5% control, *p* > 0.05). The difference between percentage of untreated cells in S phase and G2/M phase and cells treated with 100 *μ*M CAPE for 48 hours was also significant (49 ± 4.7% versus 31 ± 3.8% and 32 ± 2.9% versus 18 ± 2.6%; *p* < 0.05). The data indicated that CA and CAPE arrested Detroit 562 cells cycle after 48 hours at the G0/G1 phase in a dose- and time-dependent manner through disruption G0/G1 checkpoint, which also contributed to the growth inhibition of Detroit 562 cancer cells. This finding suggests an antiviability activity of relatively low concentrations of CAPE in malignant epithelial cells and is consistent with previous reports regarding in vitro squamous cell carcinoma cells studies.

## 4. Discussion

Head and neck cancers primarily occur in the larynx and pharynx; however, they can be also localized in oral cavity, with a predominant location on the ventral/lateral lingual site or on the floor of the mouth. Considering a relatively high risk of recurrence (20%–30%) and a low five-year survival rate (50–60%), the oncological management of these cancers has to be effective and predictable [36, 37]. Propolis and its constituents have been found to possess a cytotoxic effect on various cancer cells [38], but studies on human head and neck cancer Detroit 562 cells treated with CA/CAPE have not been reported.

The purpose of our study was to investigate the cellular response of Detroit 562 cells to two selected propolis components. Here, we demonstrated and compared the biological effects of phenolic constituents of propolis: caffeic acid and its derivative caffeic acid phenethyl ester in head and neck cancer Detroit 562 cells for the first time. Some natural substances popular in complementary medicine appear to be well suited as a potential novel agent for the adjunct treatment of certain forms of epithelial head and neck malignancy, with supportive clinical trials [39]. The results obtained from flow cytometric assay clearly revealed that CA and particularly CAPE induced dose-dependent growth inhibition and apoptosis in HNSCC, with evident alterations of Detroit 562 cell cycle. This method also identified exclusively dead cells, and CA/CAPE treatment resulted in diminishment of life of HNSCC cells. This presented study is one of the first, to the best of our knowledge, to compare the cytotoxic effects of CA and CAPE in HNSCC Detroit 562 cell line, with the conclusion that CA and CAPE moderately inhibited the proliferation and reduced the viability of HNSCC cells. These results suggest that these phenolic compounds may be potentially considered as supportive chemotherapeutic agent for certain conditions of head and neck (pre)malignancy [40]. Phenolic compounds also have been shown to alleviate the effect of chemotherapeutics in cancer cells and sequential treatment of caffeic acid and paclitaxel induces potent synergistic effect, antiproliferation, and apoptosis of lung cancer cells, which involves NF-kappa B pathway [41].

The inhibitory effect of CA/CAPE on HNSCC cells was due to its ability to induce cell cycle arrest. This is the first step to demonstrate the possibility of cell cycle perturbation by CA and CAPE on this cell line. Whereas G0/G1 arrest was induced with both CA and CAPE treatment after 48 hours of incubation, slight arrest in the S phase was induced when Detroit 562 cells were treated with CA for 24 h. Interestingly, only the concentration of 100 *μ*M of CAPE arrested mildly a Detroit 562 cell cycle in S and G2/M phase. Collectively, our results suggest that CA/CAPE inhibit head and neck cancer cell proliferation by inducing G0/G1 phase cell cycle arrest and are in agreement with other studies of contained treatment of other cancer cell lines. The G0/G1 phase can allow cells to trigger repair mechanisms or apoptotic pathways. Thus, the effects of CA and CAPE on apoptosis induction of Detroit 562 HNSCC cells were determined, and the results indicated that treatment of head and neck cancer cells with these two phenolic acids effectively induced apoptosis. Chemotherapeutic agents, including propolis constituents, are expected to inhibit the growth of some cancer cells. Apoptotic, antiproliferative and cytotoxic effects of propolis constituents have been reported previously in myeloid leukemia cells [42, 43], malignant melanoma cell line [44–46], human breast cancer cells [46–48], cervical cancer [49], and colon cancer cells [46]. A recent study reported that CAPE efficiently suppresses breast cancer stem cells from MDA-231 cells, a model of human triple-negative breast cancer [50]. Additionally, CAPE induced TRAIL-mediated cell death in Hep3B carcinoma cells [51] and stimulated the expression of death receptor 5 (DR5) and CAPE/TRAIL promoted apoptosis through the binding of TRAIL to DR5. It has been reported that CAPE inhibits proliferation [14, 15, 23, 24], COX-2 activity [52, 53], phosphoinositide 3-kinase-protein kinase B (PI3K-Akt) signaling, and Skp2-F-box protein family, responsible for downregulation of p27^Kip1^ protein [14] in human oral cancer cells. CAPE also induces apoptosis and inhibits cell growth by causing cell cycle G1 or G2/M phase arrest in different types of cancer cells [14]. Additionally, CAPE-treated human cancer cells inhibit cancer cell movement and migration [14, 54].

In particular, the current results indicate that CAPE had a greater apoptotic effect in Detroit 562 cells than did caffeic acid, which are considered the common constituents of propolis. Our findings suggest that certain doses of CA and CAPE (up to 25 *μ*M) acting for 24 hours may not affect Detroit 562 cancer cells' viability and cell cycle. Low doses of biologically active natural substances can be attributed to so-called a “hormesis effect” by even promoting cell proliferation/cell viability and this phenomenon is believed to be an adaptive response of the carcinoma cells [55]. We demonstrated that Detroit 562 HNSCC cells display variable susceptibility to CA and CAPE under different sub- and cytotoxic conditions, considering the incubation time. Kuo et al. assumed that CAPE selectively suppress human oral cancer cells due to the fact that normal human oral fibroblasts and buccal mucosal fibroblast (BF) cells were more resistant to CAPE treatment, with higher IC_50_ values [14, 56, 57]. In our study the IC_50_ for CA and CAPE treatment of Detroit 562 cells after 48 hours were 201.43 *μ*M and 83.25 *μ*M, respectively, which is coherent with the results of other studies. The IC_50_ of CAPE in cancerous human oral cell lines, to suppress proliferation of oropharyngeal squamous cell carcinoma cell line TW2.6 72.1, neck metastasis of gingiva carcinoma, tongue squamous cell carcinoma, oral squamous cell carcinoma, and oral epidermoid carcinoma-Meng 1 (OEC-M1) were 72.1 *μ*M, 101.0 *μ*M, 120.9 *μ*M, 129.7 *μ*M, and 159.2 *μ*M, respectively [14].

The anticancer activity of natural polyphenols, also present in numerous plants, fruits, and vegetables, has been extensively reported as described in preclinical studies and with regard to oral cancer, many phenolic compounds have been investigated in vitro and in vivo. Ciftci-Yilmaz et al. demonstrated that certain range of concentrations of CAPE reduces the viability of UT-SCC-74A head and neck squamous cancer stem cells [58]. According to recent study carried out by Czyżewska et al. [22] the caffeic acid induced apoptosis in 24% of the human tongue squamous cell carcinoma cell line (CAL-27) and ethanol extract of propolis, polyphenols, and mixture of polyphenolic compounds were cytotoxic for CAL-27 cells in a dose-dependent manner. EEP inhibited cell viability and induced apoptosis by upregulation of caspase-3, caspase-8, and caspase-9 in human tongue squamous cell carcinoma cell line [22]. Quercetin (flavonol, propolis ingredient) suppressed oral squamous cell proliferation by arresting G1 cell cycle phase via mitochondria-mediated apoptosis and inhibiting cell migration [40]. An inhibition of SCC-25 OSCC cells migration induced by caffeic acid was also demonstrated in oral cancer cells [26]. Some polyphenols may reverse epithelial-to-mesenchymal transition and suppress cancer invasion and in human oral cancer SCC-4 cell line [59]. To sum up, these findings indicate that caffeic acid and caffeic acid phenethyl ester could play a potential adjunct role in the therapeutic management of oral and/or head and neck cancer.

The mechanisms of activity of polyphenols comprise induction of apoptosis and cell cycle arrest, scavenging of free radicals, regulation of gene expression, and stimulation of the immune system [6–10, 60, 61]. Apoptosis plays a crucial role during oncological treatment of malignant conditions. The apoptotic range in a cell culture is a crucial parameter of cell health/viability and it can be referred to specific morphological changes. The Muse Cell Analyzer designed for a quantification of cellular apoptosis enables multidimensional cell assessment using a simplified method and does not require complicated protocols. In this study, we used the Muse Cell Analyzer for apoptosis detection using the Muse Annexin V and Dead Cell Assay. The results of available studies [62] indicate that Muse Annexin V and Dead Cell Assay allows the highly accurate assay of cellular apoptosis for both suspension and adherent cell lines using multiple treatment conditions.

Caffeic acid, CAPE, and the broad range of propolis-originated compounds are currently under scientific research and clinical investigation as a novel antitumor agents with a view at the treatment outcomes for certain types of malignancies [63]. Potentially, synergistic effects of polyphenols in propolis are responsible for their potential anticancer activities [22]. In conclusion, a combination of propolis constituents could be considered as a chemopreventive measure in a human squamous cell carcinoma originated from oral cavity or head and neck region. Due to highly individual dietary habits, populations are exposed to huge variation of bioactive natural substances present in foods. Moreover, the synergistic or additive effects of ingredients and natural compounds are responsible for the health-promoting properties of propolis-based products [30]. What is more promising, the novel technologies may enhance the therapeutic and chemopreventive potential of propolis-originated constituents, such as functionalization with nanoparticles, enhancing the efficacy of biologically active natural substances [30]. Studies explaining and clarifying the mechanisms involved in anticancer efficacy can bring invaluable data to this area of chemotherapy.

## 5. Conclusions

With limitations of in vitro study, we summarize that the current evidence of human head and neck and oral cancer adjuvant therapy and/or chemoprevention with the use of caffeic acid and/or CAPE is positive but still inconclusive. Promising results have been obtained for selected biologically active substances isolated from bee products and propolis, though the definite conclusions are still incoherent. Further advanced studies are required, following an evidence-based approach, in particular clinical trials, to confirm the clinical effectiveness of polyphenols on oral cancer treatment and prevention.
